# Colonic biopsy-associated microbial signatures are predictive of response to anti-TNFα biological therapy in Crohn’s disease

**DOI:** 10.3389/fcimb.2026.1741002

**Published:** 2026-03-04

**Authors:** Konstantina Zafeiropoulou, Ishtu L. Hageman, Tianqi Mu, Mark Davids, Andrew Y. F. Li Yim, Vincent W. Joustra, Theodorus B. M. Hakvoort, Jack Satsangi, Konstantinos Chronas, Pim J. Koelink, Manon E. Wildenberg, Rene M. van den Wijngaard, Geert R. D’Haens, Wouter J. de Jonge

**Affiliations:** 1 Tytgat Institute for Liver and Intestinal Research, Amsterdam University Medical Centers, University of Amsterdam, Amsterdam, Netherlands; 2 Amsterdam Gastroenterology Endocrinology Metabolism Institute, Amsterdam University Medical Centers, Amsterdam, Netherlands; 3 Department of Pediatric Surgery, Emma Children’s Hospital, Amsterdam University Medical Centers, University of Amsterdam, Amsterdam, Netherlands; 4 Department of Gastroenterology and Hepatology, Amsterdam University Medical Centers, University of Amsterdam, Amsterdam, Netherlands; 5 Department of Vascular Medicine, Amsterdam University Medical Centers, University of Amsterdam, Amsterdam, Netherlands; 6 Translational Gastroenterology Unit, Nuffield Department of Experimental Medicine, University of Oxford, Oxford, United Kingdom; 7 Independent Researcher, Delft, Netherlands; 8 Department of General, Visceral-, Thoracic and Vascular Surgery, University Hospital Bonn, Bonn, Germany

**Keywords:** adalimumab (ADA), adherent microbiome, biomarkers, infliximab (ifx), macrophages

## Abstract

**Introduction:**

Crohn’s disease (CD) is commonly treated with biologic therapies, including anti-TNFα agents, vedolizumab (VDZ), and ustekinumab (USTE), yet only a subset of patients respond to these treatments. This study aimed to evaluate the potential of the gut microbiome to predict treatment response.

**Methods:**

Adult CD patients initiating anti-TNFα (infliximab or adalimumab), VDZ or USTE were enrolled. Pre-treatment ileal and/or colonic biopsies were collected endoscopically. Treatment response after 26–52 weeks was defined by ≥50% reduction in the simple endoscopic score for CD and either a corticosteroid-free clinical response (≥3-point HBI decrease or remission [HBI ≤4] without systemic steroids) or a biochemical response (≥50% or ≤5 mg/L CRP reduction and ≥50% or ≤250 μg/g faecal calprotectin reduction) versus baseline. Mucosal microbiota was profiled by 16S rRNA gene sequencing of biopsies. Machine learning models predicting treatment response were trained using ASV-level count data. The impact of heat-killed bacteria on anti-TNFα–induced CD14^+^CD206^+^ macrophages was tested in mixed lymphocyte reactions (MLRs).

**Results:**

A total of 125 patients were included: 39 on anti-TNFα, 47 on VDZ, and 39 on USTE. Clinical features were similar between responders and non-responders, aside from sex (USTE-colon) and CRP (USTE-ileum). No major microbial differences were observed in VDZ, USTE ileal or colon samples. However, in colonic biopsies, anti-TNFα responders had significantly higher pre-treatment α-diversity, and 3.9% of β-diversity variation associated with response. Among six models, the anti-TNFα colonic model performed significantly better than random (AUC = 0.90) to predict response. *Mediterraneibacter gnavus* ASVs associated with non-response, whereas *Blautia* ASVs associated with response, to anti-TNFα. When tested in MLRs, pretreatment with *M. gnavus* and *B. luti* led to a reduction in macrophage polarization, with a significantly stronger effect observed for *M. gnavus* compared with *B. luti*.

**Discussion:**

Taken together, this study demonstrates that the colonic mucosal microbiome prior to anti-TNFα treatment can distinguish responders from non-responders in CD, supporting its potential as a predictive biomarker.

## Introduction

Crohn’s disease (CD) is an immune-mediated chronic inflammatory condition of the gastrointestinal tract (Andoh and Nishida, 2023). It has a multifactorial disease aetiology that is a result of an aberrant immune response towards the microbiome in a genetically susceptible host (Turpin et al., 2018). This notion is supported by clinical and experimental evidence: in CD patients, diversion of the faecal stream through surgical interventions can reduce inflammation in affected bowel segments (Burke, 2019), while in experimental animal models, intestinal inflammation is markedly more difficult to generate in germ-free conditions (Hernández-Chirlaque et al., 2016).

The faecal microbiome of CD patients is associated with an overall reduced microbial α-diversity and species richness compared to the microbiome of healthy individuals (Becker et al., 2015). The observed dysbiosis is characterized by increased prevalence of a low cell count Bacteroides 2 enterotype-like composition, with the bacterial load associating inversely with systemic and intestinal inflammation (Vieira-Silva et al., 2019).

CD patients are treated with mainstream immunosuppressive medications and biological agents such as the antibodies infliximab (IFX) and adalimumab (ADA), which both recognize TNFα (anti-TNFα), vedolizumab (VDZ), targeting the α_4_β_7_ integrin and ustekinumab (USTE) that binds the p40 subunit of interleukin (IL)-12 and IL-23 (Torres et al., 2020). Unfortunately, on average 40% of patients treated with biological agents will fail to respond or lose their response over time (Sands et al., 2004; Papamichael et al., 2015; Peyrin-Biroulet et al., 2019). Hence, clinicians have to decide upon treatments with different modes of action without a diagnostic test that can predict which therapy is suited for the individual patient. Understanding mechanisms behind treatment resistance and finding biomarkers that predict response to biological treatment would advance the current practice for CD patients (Joustra et al., 2025).

Faecal microbial signatures have been investigated for their predictive potential regarding patient response to anti-TNFα (Busquets et al., 2015; Aden et al., 2019; Ribaldone et al., 2019; Sanchis-Artero et al., 2021; Ventin-Holmberg et al., 2021; Chen et al., 2022; Park et al., 2022), VDZ (Ananthakrishnan et al., 2017) and USTE therapy (Doherty et al., 2018). However, their performance has been limited, which may be attributable to two well-established confounding factors of the faecal microbiome: diet and stool consistency (Vandeputte et al., 2016; Dinsmoor et al., 2021). It is suggested that the adherent microbiome, consisting of a bacterial community that directly adheres to the inner lining of the gut and is therefore better representative of interactions with intestinal epithelial cells and the underlying immune cells (Shi et al., 2017; Yilmaz et al., 2019; Mavragani et al., 2020; Sakurai et al., 2020; Juge, 2022), may potentially provide a more stable signature for biomarker purposes (Dovrolis et al., 2020). Likewise, the aim of this study was to assess the potential of the intestinal tissue-adherent gut microbiome to predict response of patients with Crohn’s disease to treatment with biological agents.

We collected ileal and colonic biopsies from CD patients prior to starting treatment with anti-TNFα, VDZ or USTE. Treatment response was recorded at 26–52 weeks after start of therapy, and 16S rRNA gene sequencing of biopsies was performed to assess the baseline tissue-adherent microbiome in ileum and colon. Using supervised machine learning, we identified a therapy response model based on microbial composition. The anti-TNFα colonic microbiome model outperformed randomization and showed good predictive performance. Feature importance analysis identified *Mediterraneibacter gnavus* (formerly *Ruminococcus gnavus*) ASVs associated with non-response, and *Blautia* ASVs with response. Building on our previous finding that anti-TNFα therapy response depends on the induction of CD206^+^ expressing regulatory type macrophages (Vos et al., 2011; Wildenberg et al., 2017), we performed proof-of-concept mixed lymphocyte reactions (MLRs) to explore whether *M. gnavus* and *Blautia luti* may influence macrophage polarization and, potentially, anti-TNFα responsiveness.

## Materials and methods

### Human clinical samples

We collected ileal and/or colonic intestinal biopsies from CD patients, upon baseline endoscopy, who were scheduled to start treatment with anti-TNFα, VDZ or USTE. Anti-TNFα treatment included IFX and ADA. Patients were treated according to standard-of-care protocols (Torres et al., 2020), which for VDZ meant that patients were given 300mg infusions at week 0, 2 and 6 followed by infusions at an 8 week interval, and USTE treated patients received either 260-, 390- or 520mg at week 0 with subsequent 90mg subcutaneous injections at an 8 week interval (Sandborn et al., 2013; Feagan et al., 2016). For ADA, the standard protocol was a 160mg subcutaneous induction dose, followed by 80mg 2 weeks later and thereafter 40mg injections every other week (Lichtenstein et al., 2018). Interval intensification to weekly injections were allowed, if needed at the treating physicians discretion. CD patients starting on IFX, a 5 mg/kg dose was used with standard induction at 0, 2, and 6 weeks followed by maintenance infusions every 8 weeks (Lichtenstein et al., 2018). To ensure assessment of mechanistic failures to the biological agents, only patients with measurable drug concentrations at response assessment were selected. Upon baseline endoscopy, mucosal biopsies of either ileal and/or colonic locations were taken using standard biopsy forceps. If possible, paired ileal and colonic biopsies were taken from each individual patient. All patients used bowel preparation before endoscopy consisting of macrogol and electrolytes (Klean-Prep, Norgine BV, Amsterdam, The Netherlands). The assembly of this cohort was approved by the medical ethics committee of the Academic Medical Hospital (METC NL57944.018.16 and NL53989.018.15), and written informed consent was obtained from all subjects prior to sampling. Sample sizes for each treatment group (30–40 patients) were determined based on effect sizes reported in prior microbiome studies (Busquets et al., 2015; Ananthakrishnan et al., 2017; Aden et al., 2019; Ribaldone et al., 2019; Sanchis-Artero et al., 2021; Ventin-Holmberg et al., 2021; Chen et al., 2022; Park et al., 2022; Joustra et al., 2025). Biopsies were stored in 1.10mL micronic tubes and snap-frozen immediately after sampling in liquid nitrogen.

### Defining therapy response

Response assessment occurred after 26 to 52 weeks of treatment. At response assessment, patients were defined as responders or non-responders based on endoscopic- [≥50% reduction in simple endoscopic disease activity score (SES-CD)], combined with either corticosteroid-free clinical- (≥3 point drop in Harvey Bradshaw Index (HBI) or HBI ≤4 and no systemic steroids), and/or biochemical response (C-reactive protein (CRP) and faecal calprotectin reduction ≥50% or ≤5 g/mL and faecal calprotectin ≤250 µg/g). When endoscopic assessment was not possible, imaging such as ultrasound or magnetic resonance imaging of the abdomen was used (Torres et al., 2020).

### Microbiome profiling

Microbial DNA was extracted from the intestinal biopsies. In brief, we used a combination of repeated bead beating (method 5) (Costea et al., 2017) and DNA isolation by affinity chromatography. Mechanical lysis with STAR buffer (Roche, Basel, Switzerland) was performed using FastPrep beads (BioSPX, Abcoude, The Netherlands) with three repetitive rounds of 30 s at 6.5 m/s, and with cooling for 30 s on ice in between. Finally, the DNA was obtained with the Maxwell 16 tissue Low Elution Volume total DNA purification kit (Promega, Madison, WI, USA), and DNA concentrations were measured with a Nanodrop spectrophotometer (Thermo Fisher Scientific, Bleiswijk, The Netherlands), and a Qubit fluorometric DNA quantitation method (Thermo Fisher Diagnostics, Nieuwegein, The Netherlands). Per batch of samples processed together, negative extraction controls were taken along. The DNA was used for the amplification of the bacterial 16S rRNA gene. The 16S rRNA gene amplicons were produced using a PCR procedure targeting the V3-V4 region, and this was carried out at the Microbiota Center Amsterdam (MiCA), The Netherlands. This protocol and amplification program has been published earlier (Haak et al., 2021).

Amplicon sequence variants (ASV) were extracted for each biological sample with a minimum of 4 reads (Callahan et al., 2016). Unfiltered reads were mapped against the ASV set to establish the relative abundances. Taxonomy was assigned using the IDTaxa (Murali et al., 2018) and SILVA 16S ribosomal database V132 (Quast et al., 2013). For the ASV selected during the biomarker discovery analysis, manual annotation using blast (Altschul et al., 1990) was performed, if SILVA-based annotation did not provide information at genus level. The blast search included the NCBI dataset as per February 26, 2025.

### Processing, classification and biomarker discovery analysis

Six independent machine learning models were constructed to compare responders and non-responders within the colonic and ileal sub-cohorts separately: (i) anti-TNFα – colon, (ii) anti-TNFα – ileum, (iii) VDZ – colon, (iv) VDZ – ileum, (v) USTE – colon, and (vi) USTE – ileum. All models followed a standardized four-step pipeline.

#### Step 1: filtering and stability selection

For each treatment cohort, the 250 most abundant ASVs were first selected based on mean relative abundance. Stability selection was then performed using LASSO regression (Muthukrishnan and Rohini; Haury et al., 2012; Marcos-Zambrano et al., 2021) with repeated stratified shuffle split cross-validation (50 splits). In each split, a LASSO model (α = 0.1) was fitted to the training data, and features with non-zero coefficients were recorded. Features were ranked by stability score, defined as the number of times they were selected across splits, and the top 50 stability-selected ASVs were retained for downstream analysis.

#### Step 2: supervised classification

Classification was performed using the extremely randomized trees (ExtraTrees) algorithm, a tree-based ensemble method (Geurts et al., 2006). Model development and assessment followed a combined repeated and nested cross-validation framework. For performance estimation, an outer stratified shuffle split (75% training/25% test) was repeated ten times, while hyperparameter optimization was performed within each outer loop using an inner stratified five-fold cross-validation with grid search. All preprocessing and model selection procedures were conducted exclusively on the training data within each outer repetition, where the held-out test set was used solely for final performance evaluation. Separate models were trained for colon and ileum samples to prevent cross-compartment information leakage. Cross-validation was implemented at the sample level without patient-level grouping, which represents a recognized limitation. The reported AUC values correspond to the mean performance across all outer cross-validation splits, providing a robust estimate of model generalizability.

#### Step 3: permutation testing

To assess statistical significance and overfitting, model performance was compared to randomly permuted response labels using 200 permutations per model. *P*-values were calculated as the fraction of permuted models achieving an area under the curve (AUC) equal to or greater than that of the true labels.

#### Step 4: biomarker discovery

Feature importance scores were derived from the ExtraTreesClassifier and normalized to the maximum value within each cohort, ranking ASVs by predictive power.

The entire pipeline was implemented in Python (v.3.7.7) using scikit-learn (v.0.23.1) (Pedregosa et al., 2011) and included standardized procedures for feature selection, nested cross-validation, and hypothesis testing.

### Heat-killed bacteria preparation

*M. gnavus* RJX1124 (Henke et al., 2019) and *B. luti* DSM14534 were cultured in yeast casitone fatty acids broth (YCFA) medium anaerobically at 37°C. After overnight culturing, pellets were collected and washed with RPMI supplemented with 10% fetal bovine serum (FBS, Capricorn, CP40-1314), 1% L-glutamine (L-Glu, Capricorn, CP-6174) and 1% penicillin/streptomycin (Pen/Strep, Gibco, 2585631), then resuspended in RPMI with the final optical density (OD) at 600 nm equals 10. Bacterial pellets were heat killed at 70 °C for 30 min and stored in -80°C.

### Mixed lymphocytes reactions

Human peripheral blood mononuclear cells (PBMCs) were isolated from healthy donor buffy coats (Sanquin Blood bank, Amsterdam UMC) using Ficoll density gradient centrifugation. MLRs contained PBMCs of 2 different donors, cultured in a 1: 1 ratio in RPMI (10% fetal bovine serum, 1% L-Glu, 1% Pen/strep) in U-bottom 96 well plates. After 48 hours, 10 μl/ml control human IgG (Genetex, GTX16193), or 10 μl/ml anti-TNFα (IFX) with/without heat-killed *M. gnavus* RJX1124 or *B. luti* DSM14534 (1.125 OD prior adding it to MLR) were added into MLRs and incubated for another 48 hours. Cells were collected and stained with anti CD14-PE (Bectone Dickenson 345785) and anti CD206-APC (Clone 19.2, BD Pharmingen) and analyzed using FACS Fortessa (BD) and FlowJo software (Treestar Inc., Ashland, OR).

### Statistical analysis

#### Clinical characteristics

Baseline clinical characteristics of all included patients were retrieved from Castor EDC. Statistical analyses were performed using IBM SPSS statistics (v.26.0). Differences between responders and non-responders across cohorts were assessed with the chi-square test for categorical variables and the Mann-Whitney U-test for continuous variables.

#### 16S rRNA gene sequencing

All statistical analysis of the 16S rRNA gene sequencing-derived data was performed with R (v.4.3.2, RStudio v.2023.12.1 + 402), using the phyloseq (v.1.46.0) (McMurdie and Holmes, 2013), vegan (v.2.6.4) (Oksanen et al., 2020), and stats (v.4.3.2) packages. Alpha diversity was examined at observed species richness, Shannon index, Simpson index and Fisher’s alpha using the count table at ASV level, and compared between responders and non-responders groups of patients using Wilcoxon signed-rank test. Microbial composition was assessed using principal coordinate analysis (PCoA) at the ASV level based on Bray-Curtis dissimilarity index (BCD), weighted (WUD) and unweighted UniFrac (UUD) distance matrices. The former considers bacterial taxon abundance, whereas the two latter consider phylogenetic distance between bacterial taxa through presence/absence. Permutational multivariate analysis of variance (PERMANOVA) was applied using the vegan *adonis* function. To assess the influence of prior biologic exposure on microbial community structure, environmental fitting was performed using the *envfit* function in the vegan package, projecting variables corresponding to earlier biologic therapy (pre-anti-TNF, pre-VDZ, pre-USTE) onto the PCoA ordination space. Group centroids were calculated for each treatment category by pooling colonic and ileal biopsies. Euclidean distances between centroids were computed from the first two PCoA axes using the *dist* function in the R stats package to quantify the magnitude of microbiome shifts associated with treatment history, while vectors were used to visualize the direction of these shifts within the ordination space. Differences in the relative abundance of ASV-of-interest between responders and non-responders were performed using Mann-Whitney U test.

#### Machine learning

Overfitting of the extra trees-based models was evaluated by permuting the labels 200 times and calculating the probability of randomly achieving an AUC equal to or greater than that of the extra trees-based model.

#### Mixed lymphocyte reactions

Differences between *M. gnavus* RJX1124 and *B. luti* were assessed using Student’s t-test for normally distributed data or Mann-Whitney U test for non-normally distributed data, performed in GraphPad Prism (v.10.2.0).

## Results

### Baseline characteristics

**Figure 1:**
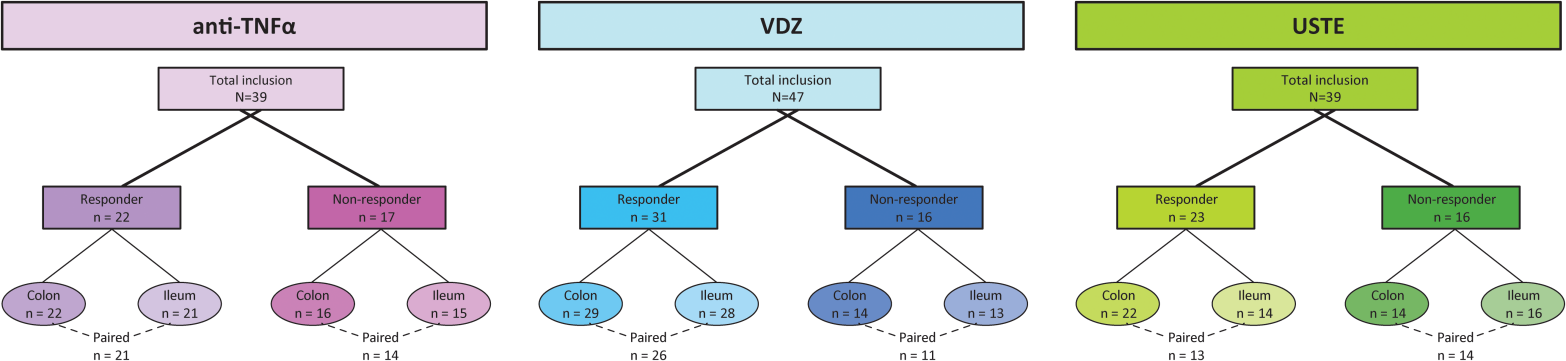
Distribution of patients across biologic treatments: anti-TNFα, vedolizumab (VDZ), and ustekinumab (USTE). Baseline biopsies were obtained from the ileum and/or colon. Dashed lines indicate patients with paired colonic and ileal biopsies.

The anti-TNFα cohort consisted of 39 patients who started treatment with IFX or ADA (**Figure 1**). Colonic biopsies were collected from 22 responders and 16 non-responders, while ileal biopsies were collected from 21 responders and 15 non-responders. The clinical characteristics of both sub-cohorts were similar (**Table 1**). Paired ileal and colonic biopsies could be obtained from 21 responders and 14 non-responders.

**Table 1:** Baseline clinical characteristics of patients treated with anti-TNFα.

	COLON			ILEUM		
	Responder N = 22	Non-responder N = 16	*P*	Responder N = 21	Non-responder N = 15	*P*
Biological agent, n (%) - Infliximab	9 (40.9)	6 (37.5)	.83	9 (42.9)	6 (40.0)	0.87
Sex, female, n (%)	13 (59.1)	10 (62.5)	.83	12 (57.1)	8 (53.3)	0.82
Age (years)	31.5 (20.8-40.3)	26.0 (22.3-35.3)	.58	32.0 (23.0-40.5)	25.0 (22.0-33.0)	0.22
Ethnic background, n (%) - Caucasian	18 (81.8)	12 (75.0)	.61	17 (81.0)	11 (73.3)	0.59
Diet, no restrictions, n (%)	19 (86.4)	12 (75.0)	.37	18 (85.7)	11 (78.6) [1]	0.58
Family history of IBD, n (%)	2 (9.5) [1]	1 (6.7) [1]	.76	2 (10.0) [1]	1 (7.7) [2]	0.82
C-reactive protein (mg/L)	2.7 (1-17.8)	8.0 (1.6-14.3)	.56	2.3 (1.0-13.0)	8.1 (1.6-13.1)	0.40
Fecal calprotectin (ug/g)	577 (67-1,704) [3]	924 (925-2,035) [2]	.23	488 (61.8-488) [3]	914 (382-1,452) [2]	0.23
Total HBI	4.0 (3.0-7.0) [1]	6.0 (2.5-9.0) [3]	.60	4.5 (3.0-7.0) [1]	5.0 (2.0-9.0) [3]	0.95
Total SES-CD	7.0 (4.0-12.3)	8.5 (5.5-13.0) [2]	.79	7.0 (4.0-12.5)	9.0 (6.8-13.0) [1]	0.65
Disease location, n (%) - Ileal disease (L1) - Colonic disease (L2) - Ileocolonic disease (L3) - Upper GI involvement (L4)	4 (18.2) 6 (27.3) 12 (54.5) 0 (0.0)	6 (37.5) 2 (12.5) 8 (50.0) 0 (0.0)	.32	4 (19.1) 5 (23.8) 12 (57.1) 0 (0.0)	5 (33.3) 2 (13.3) 8 (53.3) 0 (0.0)	0.54
Disease behavior, n (%) - Non structuring non-penetrating (B1) - Stricturing (B2) - Penetrating (B3) - Perianal disease (p)	14 (63.6) 2 (9.1) 6 (27.3) 8 (36.4)	10 (62.5) 3 (18.8) 3 (18.8) 5 (31.3)	.62	13 (61.9) 2 (9.5) 6 (28.6) 8 (38.1)	10 (66.7) 2 (13.3) 2 (13.3) 4 (26.7)	0.48
Previous IBD surgery, n (%)	13 (59.1)	8 (53.3) [1]	.47	13 (61.9)	8 (57.1) [1]	0.46
Concomitant medication, n (%) - Immunomodulators	11 (50.0)	7 (43.8)	.70	10 (47.6)	6 (40.0)	0.65
Previous biological treatment exposure, n (%) - Anti-TNFα - Vedolizumab - Ustekinumab	13 (59.1) 9 (40.9) 3 (13.6) 2 (9.1)	11 (68.8) 10 (62.5) 2 (12.5) 1 (6.3)	.54 .19 .92 .75	12 (57.1) 9 (42.9) 3 (14.3) 2 (9.5)	11 (73.3) 10 (66.7) 3 (20.0) 2 (13.3)	0.32 0.16 0.65 0.72
Smoking, active, n (%)	3 (13.6)	5 (31.3)	.37	3 (14.3)	5 (33.3)	0.12

Values are median (interquartile range) unless otherwise defined. The number of missing data is shown in square brackets. Percentages have been calculated in the available data. Anti-TNFα: infliximab & adalimumab; HBI, Harvey Bradshaw Index; SES-CD, simple endoscopic disease activity score; Immunomodulator: azathioprine, mercaptopurine, thioguanine, methotrexate.

The VDZ cohort comprised 47 patients (**Figure 1**). Colonic biopsies were collected from 29 responders and 14 non-responders, and ileal biopsies from 28 responders and 13 non-responders, with similar clinical characteristics (**Supplementary Table S1**). Paired ileal and colonic biopsies were obtained from 26 responders and 11 non-responders.

Lastly, the USTE cohort included 39 patients (**Figure 1**). Colonic biopsies were collected from 22 responders and 14 non-responders, with similar clinical characteristics, except for sex, where a higher proportion of females was observed in the responders group (90.9%) compared to the non-responders group (64.3%, *P* = 0.05, **Supplementary Table S2**). Ileal biopsies were collected from 14 responders and 16 non-responders, showing no sex differences, but significant differences in CRP levels (median [interquartile range]): non-responders had higher levels (7.8 [2.9-17.5] mg/L) compared to responders (2.0 [0.8-3.6] mg/L, *P* = 0.004, **Supplementary Table S2**). Paired ileal and colonic biopsies were obtained from 13 responders and 14 non-responders.

In comparison to the anti-TNFα cohort (colonic responders 59.1% *vs.* non-responders 68.8%; ileal responders 57.1% *vs.* non-responders 73.3%, **Table 1**), earlier treatment with biological agents was notably higher in the VDZ (colonic responders 62.1% *vs.* non-responders 85.7%; ileal responders 60.7% *vs.* non-responders 84.6%, **Supplementary Table S1**) and USTE cohort (colonic responders 95.5% *vs.* non-responders 92.9%; ileal responders 100% *vs.* non-responders 93.8%, **Supplementary Table S2**).

### Mucus-associated adherent microbiome composition prior to treatment

In total, 6,174 unique ASVs were identified across all samples (**Supplementary Table S3**). Of these, 343 ASVs (5.6% of the total) were annotated to host-associated eukaryotes or of unknown origin (**Supplementary Table S4**), and collectively accounted for an average of 9.3% of the total microbiome per sample (mean ± standard deviation: 9.3 ± 16.6%). These ASVs were excluded from downstream analyses.

**Figure 2:**
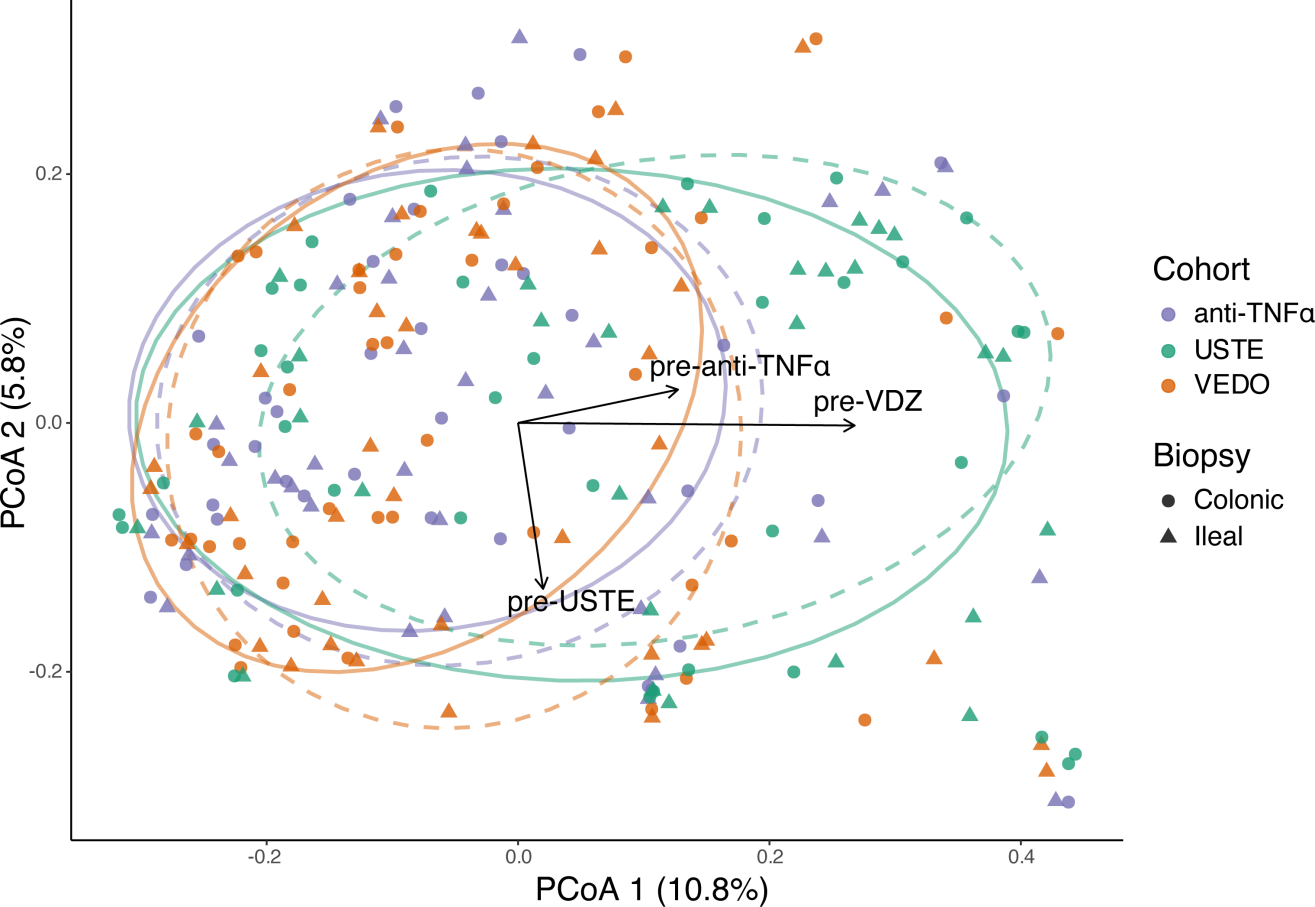
Principal coordinates analysis (PCoA) of Bray-Curtis dissimilarities showing microbiome composition across treatment groups. Samples are coloured by intervention (anti-TNFα, VDZ, USTE) and shaped by biopsy location (colon, ileum). Ellipses indicate the 68% confidence interval around group centroids; solid ellipses represent colonic biopsies, whereas dashed ellipses represent ileal biopsies. Arrows represent centroid vectors corresponding to prior biologic exposure (pre-anti-TNFα, pre-VDZ, pre-USTE), illustrating the direction of microbiome shifts associated with treatment history within the ordination space.

Since prior treatment with biologic agents was more frequent in the VDZ and USTE cohorts, we first evaluated its impact on overall microbial composition. Euclidean distances between the centroids of the three intervention groups (anti-TNFα, VDZ, USTE) were calculated (**Supplementary Table S5**), and prior biologic exposure was visualized on PCoA plots using vectors (**Figure 2**). Notably, VDZ samples shifted downward along PCoA2 relative to anti-TNFα, aligning with the vector of prior-USTE treatment, while USTE samples shifted right along PCoA1, following the vector of prior-VDZ exposure. Compared to anti-TNFα, the cohort with the least prior biologic exposure, centroid differences were moderate for VDZ (0.018) and substantial for USTE (0.110), suggesting that prior biologic therapy significantly modifies the microbiome.

**Figure 3:**
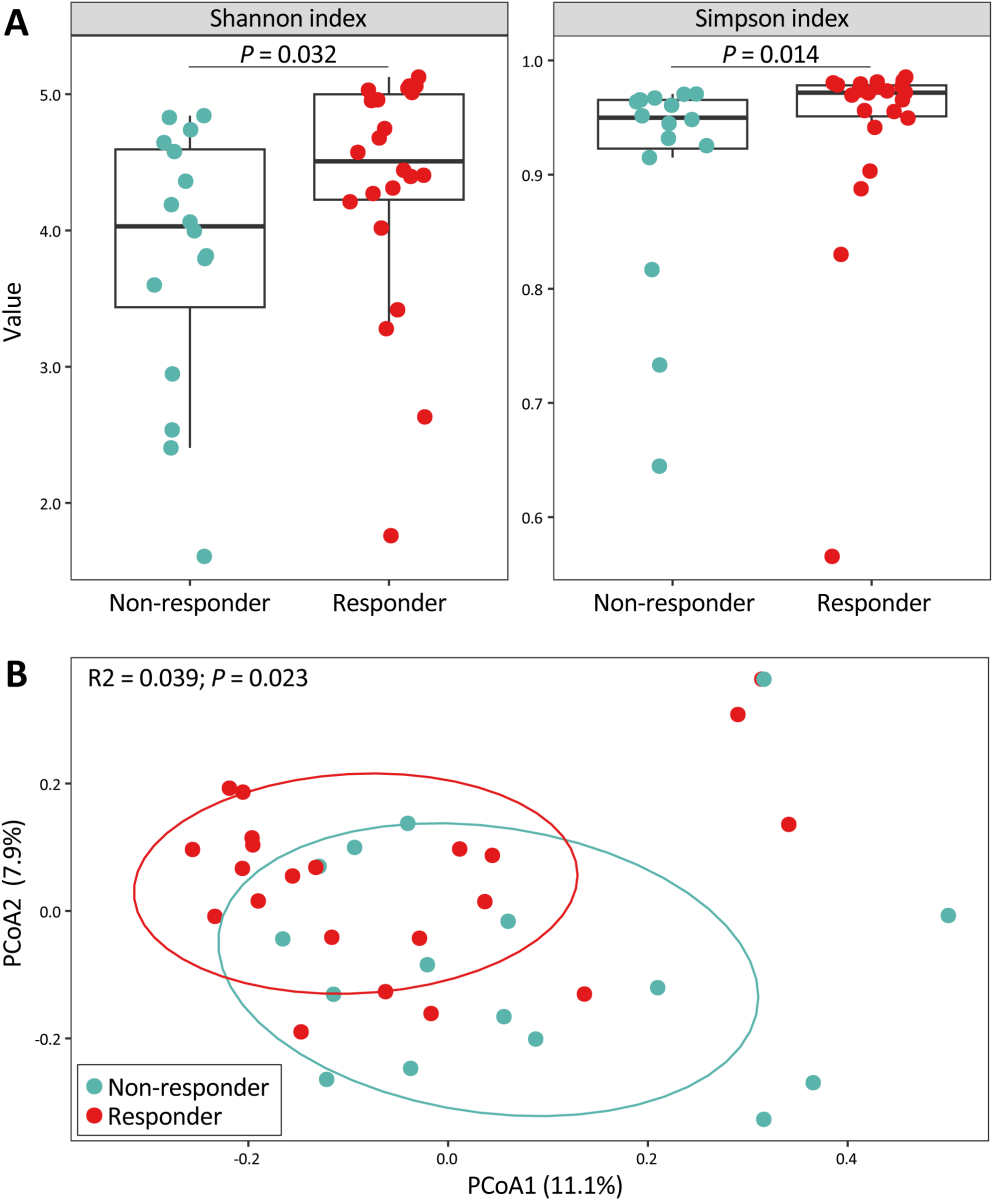
Baseline colonic mucosal-adherent microbiome in patients treated with anti-TNFα. **(A)** Alpha diversity measured by Shannon and Simpson indices at amplicon sequence variant (ASV) level. Wilcoxon signed-rank test. **(B)** Principal coordinate analysis (PCoA) based on Bray–Curtis dissimilarity at ASV level, illustrating beta diversity between responders and non-responders. Permutational multivariate analysis of variance (PERMANOVA).

Within-sample α-diversity metrics showed substantial overlap between responders and non-responders across most sub-cohorts (**Supplementary Table S6**). The exception was observed in colonic biopsies from patients initiating anti-TNFα therapy, where responders exhibited higher Shannon and Simpson indices compared with non-responders (*P* = 0.032 and 0.014, respectively, **Figure 3A**). Medication-specific analyses indicated that this difference was primarily driven by ADA-treated patients, whereas no statistically significant differences were observed in IFX-treated patients, likely reflecting limited statistical power (**Supplementary Figure S1**).

Consistent with α-diversity, most responders and non-responders overlapped on PCoA plots across sub-cohorts (**Supplementary Table S7**), with the exception of colonic biopsies from anti-TNFα patients. In the pooled anti-TNFα cohort, response status explained a limited proportion of variance in colonic mucus-associated adherent microbiota composition (Bray-Curtis R^2^ = 0.039; *P* = 0.023; **Figure 3B**), indicating modest compositional differences prior to treatment initiation. Medication-specific analyses revealed comparable effect sizes for ADA (R^2^ = 0.065) and IFX (R^2^ = 0.060) patients, although statistical significance was reached only in ADA (*P* = 0.036), likely reflecting differences in sample size and statistical power (**Supplementary Figure S1**).

### Machine learning-based prediction of the response to treatment

**Figure 4:**
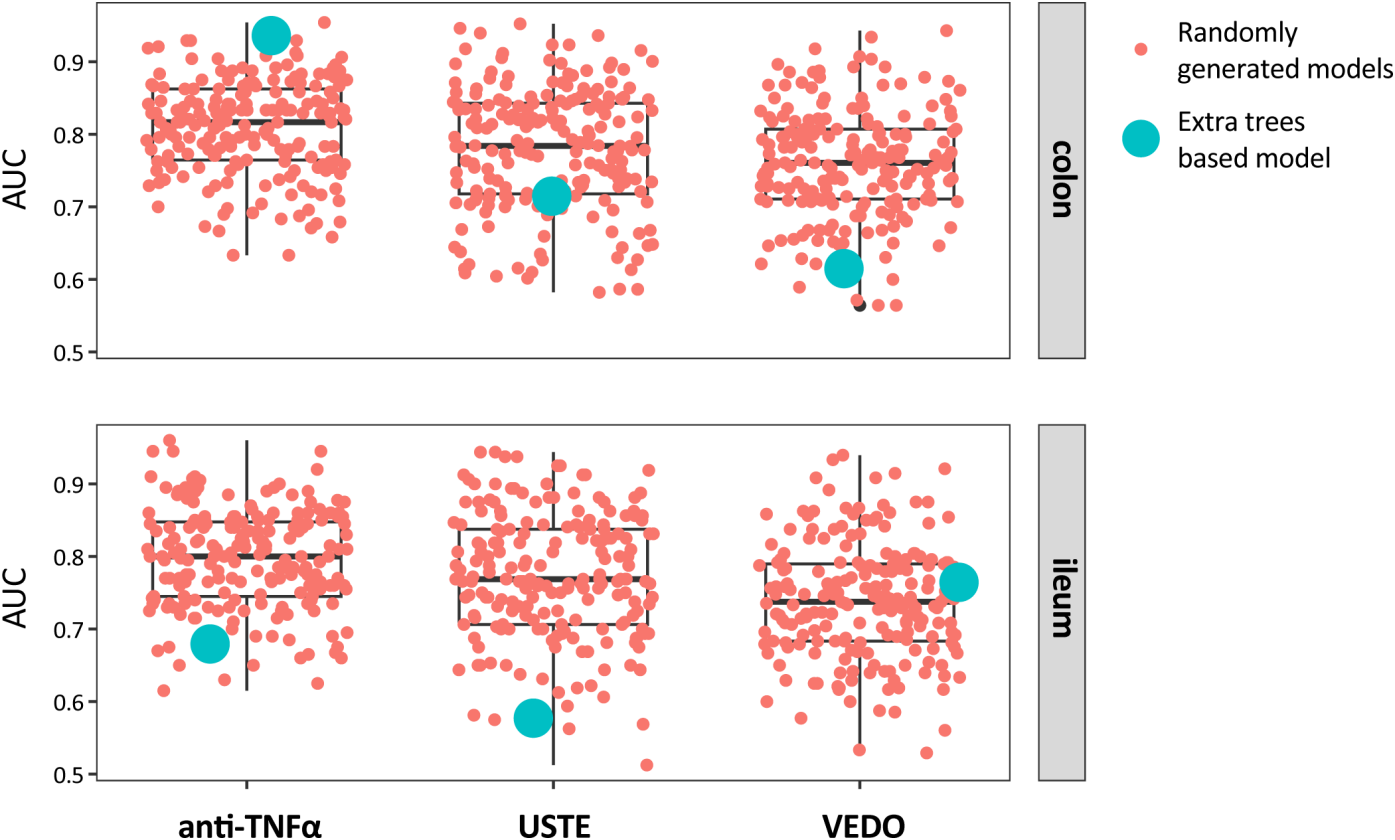
Prediction of treatment response using extra trees-based and random models across biological therapies: anti-TNFα, vedolizumab (VDZ), and ustekinumab (USTE). Models were trained on baseline colonic (top panel) or ileal (bottom panel) mucosal-adherent microbiome profiles at the ASV level.

**Figure 5:**
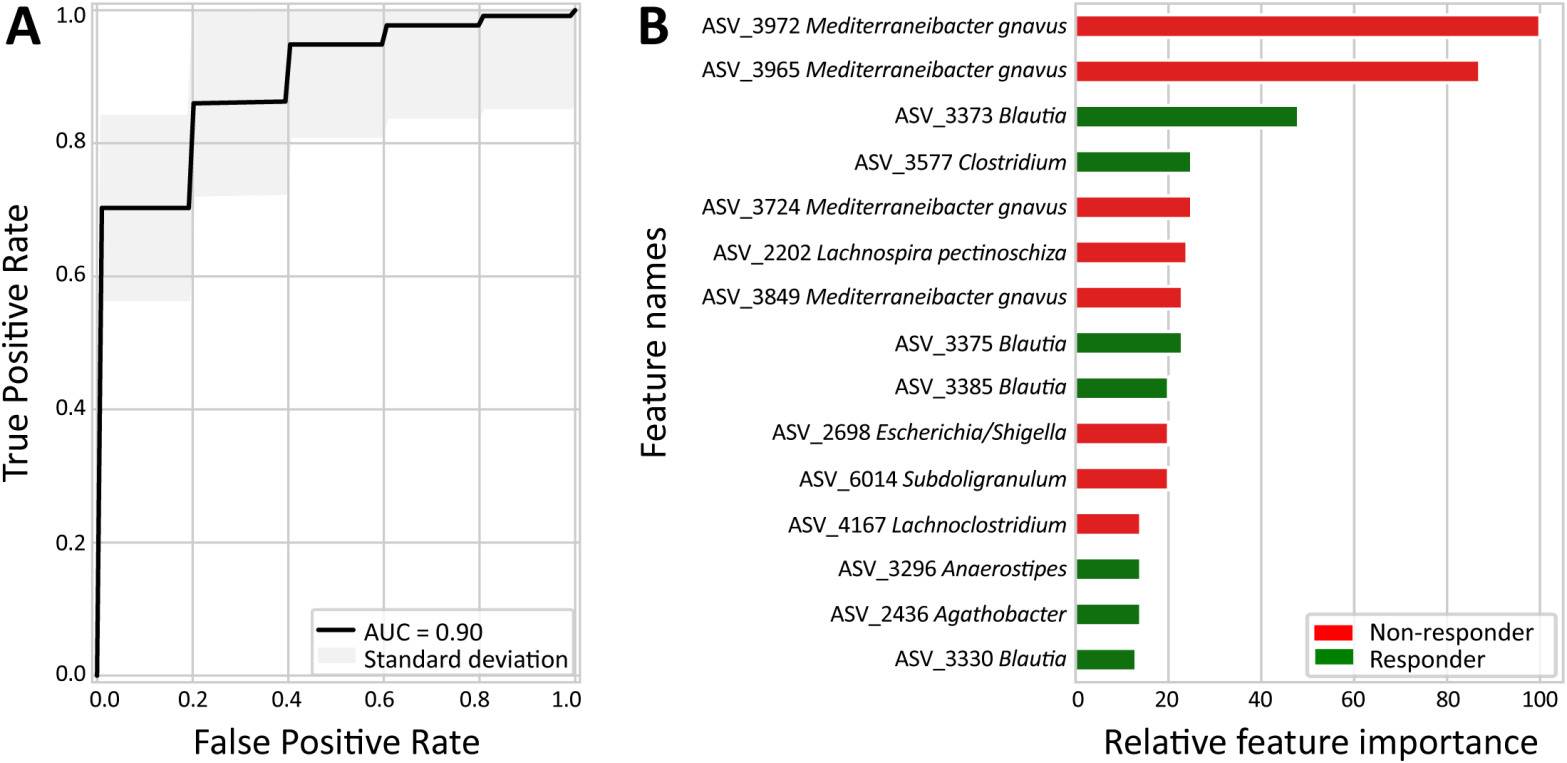
Extra trees-based model predicting anti-TNFα treatment response using the colonic mucosal-adherent microbiome. **(A)** Receiver operating characteristic (ROC) curve of the extra trees-based model trained on baseline colonic microbiome profiles. Area under the curve (AUC). **(B)** Top 15 most predictive ASVs identified by the extra trees-based model, ranked by relative feature importance (x-axis). Bar colour indicates association with response (green) or non-response (red).

A total of six distinct models were generated to explore whether the pre-treatment mucus-associated adherent microbiome of the colonic and/or ileal biopsies of the patients could predict their response to the respective treatment: (i) anti-TNFα - colon, (ii) anti-TNFα – ileum, (iii) VDZ – colon, (iv) VDZ – ileum, (v) USTE – colon, and (vi) USTE – ileum. Five of the six models failed to predict response better than the randomly generated models, where response labels were permuted 200 times per model (*P* >.05, **Supplementary Table S8**), except for the colon-anti-TNFα cohort (**Figure 4**). The machine learning-based model using the mucus-associated adherent microbiome of the colonic biopsies of patients who started treatment with anti-TNFα could predict significantly better than the randomly generated models the response to the anti-TNFα treatment, with a good predictive performance of AUC = 0.90 (*P* = 0.005, **Figure 5A**).

### Biomarker discovery analysis for therapy with anti-TNFα

In the acquisition of our machine learning-based models, feature importance scores were used to determine the relative importance of each stability-selected ASV when building a predictive model. In the colonic sub-cohort of patients that started treatment with anti-TNFα, the feature importance values of the 15 most informative ASVs in predicting response to anti-TNFα ranged from 0.009 to 0.068 (**Supplementary Table S9**). Visualization of the feature importance values relative to the highest value (here, 0.068) are shown in **Figure 5B**. Eight out of the 15 most informative ASVs were more abundant in the non-responders to anti-TNFα patients, including four ASVs identified as *M. gnavus* (ASV_3972, ASV_3965, ASV_3724, and ASV_3849), one ASV each of *Lachnospira pectinoschiza* (ASV_2202)*, Escherichia*/*Shigella* (ASV_2698, 16S does not allow us to differentiate these two taxa), *Subdoligranulum* (ASV_6014), and *Lachnoclostridium* (ASV_4167) (all shown in red in **Figure 5B**). The remaining seven ASVs were associated with anti-TNFα therapy response, including four ASVs of the *Blautia* genus (ASV_3373, ASV_3375, ASV_3385, ASV_3330), and one ASV each of *Clostridium scindens* (ASV_3577)*, Anaerostipes* (ASV_3296), and *Agathobacter* (ASV_2436) (all shown in green in **Figure 5B**).

Stratification by anti-TNFα agent revealed that two of the four ASVs annotated to *M. gnavus* (ASV_3972 and ASV_3965) were consistently more abundant in baseline colonic biopsies of non-responders compared with responders to both ADA and IFX (**Supplementary Figure S2**). While this difference did not reach statistical significance for IFX, likely reflecting the limited sample size, the concordant direction of effect across therapies reinforces *M. gnavus* as a key microbial feature associated with anti-TNFα response and supports the combined analysis of anti-TNFα treatments in this study.

### *M. gnavus* negates M2-polarization

Previous research has shown that the differentiation of CD14^+^ monocytes to CD206^+^ M2-like regulatory macrophages is associated with anti-TNFα therapy response in IBD (Vos et al., 2011; Wildenberg et al., 2017). Among the taxa identified through our biomarker analysis, *M. gnavus* and *Blautia* emerged as the most prominent microbial markers linked to anti-TNFα treatment outcome; *M. gnavus* with four ASVs (ASV_3972, ASV_3965, ASV_3724, and ASV_3849) associated with non-response, and *Blautia* with four ASVs (ASV_3373, ASV_3375, ASV_3385, and ASV_3330) associated with response. We therefore sought to mechanistically assess whether these taxa modulate anti-TNFα-induced M2 polarization. We hypothesized that *M. gnavus* negatively interferes with anti-TNFα-driven M2 differentiation, which we tested using the *M. gnavus* RJX1124 strain isolated from an IBD patient biopsy (Henke et al., 2019). Conversely, we postulated that *Blautia* promotes anti-TNFα-induced M2 polarization. Because ASV-based single-species identification within this genus was ambiguous, we employed *Blautia luti*, one of the most abundant *Blautia* species in the human intestine (Liu et al., 2021), as a representative strain.

**Figure 6:**
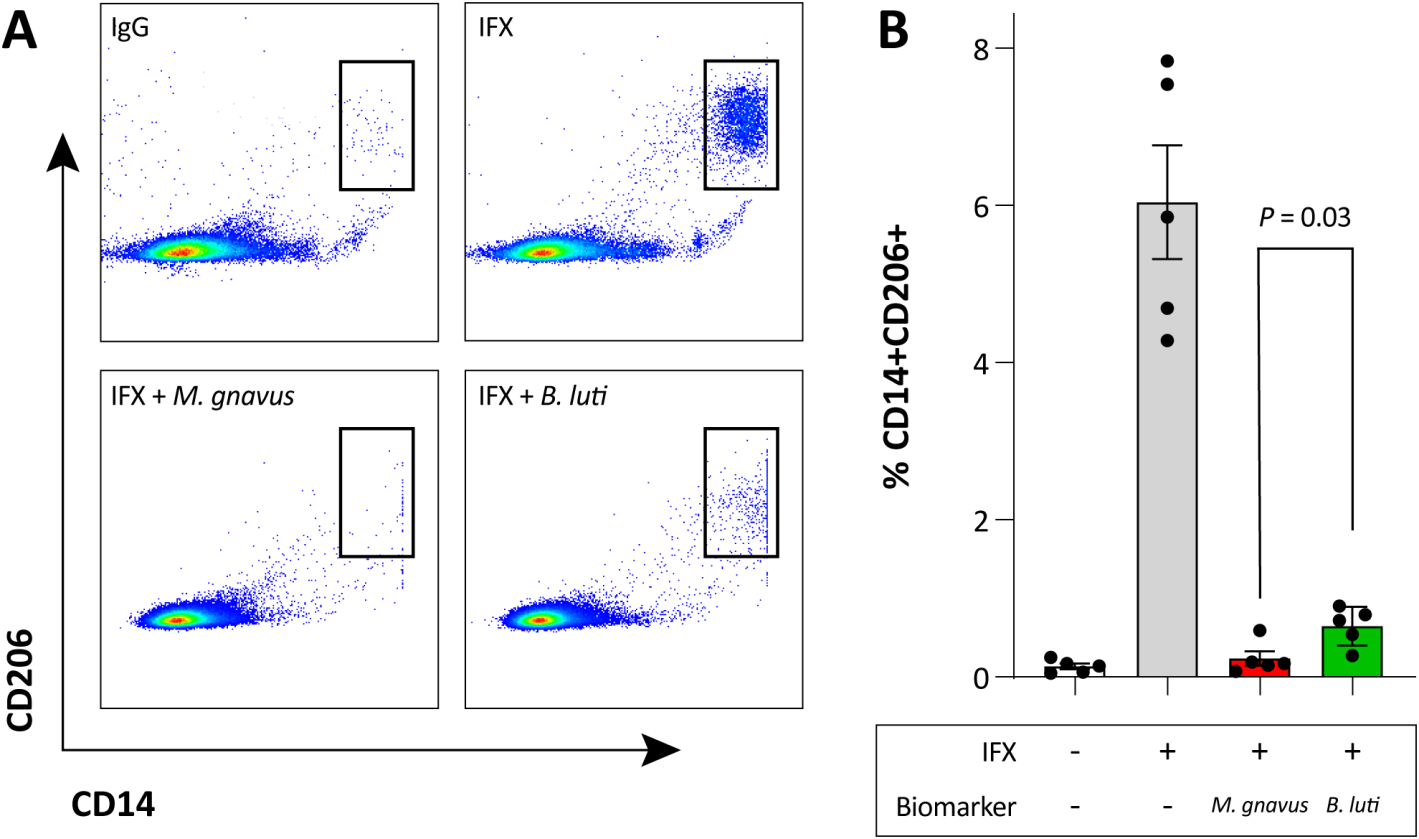
Differential induction of M2 macrophages by representative strains associated with anti-TNFα treatment response. **(A)** Flow cytometry plots showing CD14^+^CD206^+^ M2 macrophages in mixed lymphocyte reactions in the presence of IgG (top left), infliximab (IFX, top right), heat-killed *Mediterraneibacter gnavus* RJX1124 (bottom left), or *Blautia luti* (bottom right). Dot plots represent total cultures; histograms are gated for CD14^+^CD206^+^ cells. Data are from one representative experiment of three, performed using cells from two individual donors. **(B)** Quantification of CD14^+^CD206^+^ cells, shown as mean ± standard deviation. Bar colour indicates association with response (green, *B*. *luti*) or non-response (red, *M. gnavus*).

In agreement with our previous studies, IFX showed successful induction of CD14^+^CD206^+^ macrophages, compared to IgG control antibody (**Figure 6A**). In the presence of *M. gnavus* the M2-polarizing effect of IFX was almost completely negated. Notably, *B. luti* also showed a considerable negative effect on macrophage polarization towards CD206. However, the percentage of CD14^+^CD206^+^ macrophages was still significantly higher when compared to *M. gnavus* MLRs (*P* = 0.03, **Figure 6B**). Taken together, the presence of *M. gnavus* negatively affects the potential of anti-TNFα to induce regulatory macrophages.

## Discussion

In this study, we demonstrate that the intestinal tissue-adherent microbiome obtained from diagnostic colonic biopsies prior to treatment initiation contains informative signatures associated with response to anti-TNFα therapy in Crohn’s disease. By profiling pre-therapy intestinal biopsies using 16S rRNA gene sequencing, we characterized baseline mucosa-associated microbial communities and leveraged these data to identify microbial features linked to treatment outcome. Using machine learning techniques incorporating stability selection and extra trees classifier, we identified a robust panel of ASVs with high discriminatory performance for classifying responders and non-responders to anti-TNFα therapy based solely on baseline colonic microbiota profiles. Importantly, these associations were observed prior to treatment initiation, highlighting the potential clinical relevance of tissue-adherent microbial signatures for stratifying patients before exposure to biological agents. To explore potential biological mechanisms underlying these associations, we performed a proof-of-concept anti-TNFα macrophage polarization experiment. In this setting, the presence of *M. gnavus* was associated with reduced numbers of M2-like macrophages, supporting a functional link between specific microbial taxa and host immune responses relevant to anti-TNFα efficacy. Collectively, our findings indicate that the colonic mucosa-associated microbiota represents a promising source of baseline biomarkers for anti-TNFα therapy in CD and suggest that *M. gnavus* may contribute to microbial-immune interactions associated with treatment non-response.

Most microbiome studies to date have relied on faecal samples because they are easily accessible. While some of these investigations have incorporated machine learning approaches, predictive performance has generally been moderate and often lacks validation across independent training-test splits (Ventin-Holmberg et al., 2021). When appropriately processed, intestinal biopsies have been shown to comprise both loosely-adherent and strictly-adherent microbial communities (Mukhopadhya et al., 2022), which more closely reflect the physiological environment of the gut mucosa, including mucus secretion and anti-microbial peptide secretion, and show less diet induced variability (Zoetendal et al., 2002; Vaga et al., 2020). Moreover, analysis of tissue-adherent microbiota circumvents well-established confounders associated with faecal sampling, such as stool consistency (Vandeputte et al., 2016). Based on these considerations, we hypothesized that tissue-adherent bacteria may represent more stable biomarkers than faecal microbiota and therefore provide improved prediction of medication response.

Indeed, for anti-TNFα our approach demonstrated good predictive performance for colonic biopsies. However, prediction using ileal biopsies did not exceed performance expected by chance, that is, permuted labels. This discrepancy may relate to α-diversity, which was significantly higher in colonic biopsies from responders than non-responders, a pattern not observed in the ileum. We speculate that a relatively rich adherent microbiota in the colon may support treatment response in CD. Nevertheless, the primary aim of this study was to use machine learning to identify predictive microbiome features rather than to compare microbial composition using conventional metrics. Accordingly, interpretations based on α- and β-diversity should be approached with caution.

Our approach was unable to provide accurate prediction of response to VDZ or USTE, which we attribute to prior exposure to biologics. Nearly 50% of patients receiving anti-TNFα were biologic-naïve, whereas most patients treated with VDZ and USTE had undergone multiple rounds of biologic therapy. We found that prior exposure to VDZ or USTE shifted the adherent microbial composition, driving it toward a more “dysbiotic” state and thereby limiting the predictive value of post-treatment microbiome profiles. Overall, these results suggest that microbiome-based prediction may be less feasible in heavily pretreated patient populations.

We focused on ASVs profiled before treatment initiation. ASVs linked to *M. gnavus* and *Blautia* emerged as the strongest predictors of treatment outcome, associated with non-response and response to anti-TNFα, respectively. These findings align with previous studies showing that *M. gnavus* is enriched in baseline samples of non-responders and decreases following successful ADA therapy in Crohn’s disease patients (Ribaldone et al., 2019). Conversely, *Blautia* has been consistently associated with response to IFX (Zhuang et al., 2020), and is more abundant in pre-treatment samples of responders (Park et al., 2022).

*M. gnavus* is a key indicator of a low-diversity, dysbiotic “Bacteroides2-like” enterotype composition (Bresser et al., 2022), which is often observed in patients with CD (Vieira-Silva et al., 2019). Several studies have elucidated mechanisms relevant to *M. gnavus*’ s role in human health and disease (Crost et al., 2023). For one, *M. gnavus* demonstrates the ability to adapt and adhere to the gut lining; it is known to produce antimicrobial peptides, including bacteriocins to kill other bacterial taxa (Crost et al., 2023). Furthermore, it can use complex carbohydrates as nutrients and degrade intestinal mucus for its own energy source (Hall et al., 2017; Franzin et al., 2021; Crost et al., 2023). In IBD, it is hypothesized that aberrant mucus degradation might lead to increased gut permeability, possibly enhancing the inflammatory response (Hall et al., 2017). *M. gnavus* also harbours glucorhamnan on the cell surface, a polysaccharide that potently induces TNFα secretion by dendritic cells, in a toll-like receptor 4-dependent manner (Henke et al., 2019). Thus, in CD patients with high relative abundance of *M. gnavus*, i.e. anti-TNFα non-responders, glucorhamnan may play a role in uncontrolled secretion of TNFα. This could partially explain why anti-TNFα is inefficacious in these patients and warrants future investigations into this polysaccharide and its possible relation to treatment failure. Besides glucorhamnan, some *M. gnavus* strains harbour another, recently described, capsular polysaccharide that promotes a tolerogenic instead of a proinflammatory immune response. Henke et al. showed that the absence of this protective polysaccharide, as observed in some IBD-derived isolates, elicited a robust inflammatory immune response (Henke et al., 2021). Lack of this capsular *M. gnavus* polysaccharide could also be important in response’ s failure. The possible relevance of the two different *M. gnavus* expressed polysaccharides in therapy response can be addressed by *in vitro* assays, for which tissue adherent strains will have to be isolated from an anti TNFα treatment cohort.

To investigate whether ASVs annotated to *M. gnavus* and *Blautia* may influence anti-TNFα responsiveness, we performed a series of *in vitro* proof-of-concept experiments. We focused on anti-TNFα-induced polarization of macrophages toward the M2 phenotype, which is known for its anti-inflammatory properties and role in tissue repair. The balance between (pro-inflammatory)-M1 and (anti-inflammatory)-M2 macrophages is critical for the development and progression of CD (Zhou et al., 2019; Zhang et al., 2023). In previous mouse transfer colitis studies, we observed that anti-TNFα therapy induces a shift toward CD14^+^CD206^+^ M2 regulatory macrophages (Wildenberg et al., 2017). This shift was confirmed *in vitro* using both mouse and human MLR assays and corroborated in a small *in situ* study of anti-TNFα treated patients (Vos et al., 2011; Wildenberg et al., 2017), where induction of regulatory macrophages occurred only in successfully treated patients.

Here, we assessed the effect of *M. gnavus* and *B. luti* on anti-TNFα induced M2 macrophage polarization in human MLRs. Because fresh biopsies were not available, isolation of bacterial strains from patient biopsies was not feasible; therefore, these experiments were restricted to the strains *M. gnavus* RJX1124 (Henke et al., 2019) and *B. luti* DSM14534. The *M. gnavus* RJX1124 strain was selected because it was originally isolated from an IBD biopsy. However, this strain is characterized by the absence of a tolerogenic polysaccharide, expression of pro-inflammatory glucorhamnan, and a strong capacity to induce TNFα production in murine bone-marrow-derived dendritic cells (Henke et al., 2021). These functional properties cannot be inferred from our ASV-level resolution, and the strains driving the predictive associations in our models may therefore differ, potentially leading to different outcomes in the MLR assay. The *B. luti* DSM14534 strain was selected in an even more arbitrary manner. In contrast to *M. gnavus*, for which BLAST-based querying allowed species-level annotation of associated ASVs, ASVs assigned to *Blautia* could not be resolved to the species level using either the SILVA database or NCBI BLAST searches. Consequently, strain selection could not be guided by sequence-based annotation. We therefore selected *B. luti* based on species-level relevance, as it represents one of the most abundant *Blautia* species in the human gut microbiome (Liu et al., 2021), providing a biologically plausible proof-of-concept model. However, this approach inherently implies that alternative *Blautia* species or strains could exert different immunomodulatory effects, and that the outcome of the MLR assays may vary accordingly.

The successful induction of CD14^+^CD206^+^ regulatory macrophages by IFX was completely reversed by the addition of heat killed *M. gnavus*. This result may explain negative treatment outcome in patients with high pre-therapy level of adherend *M. gnavus*. Yet, it has to be acknowledged that *B. luti* also diminished, albeit to a lesser extent, the polarizing effect of IFX. As mentioned above, this may relate to the difficulty of identifying which *Blautia* spp. are relevant in our predictions for therapy response. Possibly other species of the *Blautia* genus would have given a different, i.e. less inhibiting, response. Better identification on species level could become relevant for future therapy as well. One can envision, that identification of the exact species contributing to therapy success may eventually lead to a probiotic-like add on treatment for anti-TNFα therapy.

Our study has several strengths. CD patients were rigorously classified as responders or non-responders based on stringent clinical, endoscopic, and biochemical criteria. We focused on adherent microbial signatures from intestinal biopsies rather than faecal samples, which are often suboptimal for capturing mucosa-associated microbial dynamics. While intestinal biopsies are inherently low in biomass and therefore more susceptible to contamination during sampling, storage, DNA extraction, and amplification, we implemented a comprehensive decontamination screening protocol to ensure high-quality input for both conventional and machine learning analyses. Additionally, working at the ASV level, rather than the broader genus level, enabled the detection of more specific microbial signals, such as *M. gnavus*, which appeared in four of the 15 most informative ASVs within the anti-TNFα colonic cohort. Importantly, our predictive ASVs were identified in colonic samples from anti-TNFα–treated patients regardless of disease location (ileal, colonic, or ileocolonic CD), indicating that the predictive model is robust across different CD subtypes.

One primary limitation of our approach is that the anti-TNFα patient cohort included individuals treated with two different biologicals, ADA and IFX. This choice was guided by several considerations: (a) both ADA and IFX target TNFα; (b) sample sizes for ADA and IFX separately were too small to support machine learning analyses, which were the primary focus of this study; and (c) α-diversity analyses within the ADA and IFX subgroups showed that responders to both therapies tended to have higher α-diversity, although the difference was not significant for IFX, likely due to limited sample size. Despite structural differences (IFX is a humanized mouse monoclonal antibody, whereas ADA is fully human (Mpofu et al., 2005)), we have previously demonstrated that both antibodies similarly induce macrophage polarization and inhibit T-cell proliferation (Vos et al., 2011). In the current study, the most informative ASVs predicting non-response to anti-TNFα showed higher relative abundance in non-responders compared with responders in both the ADA and IFX subgroups, further supporting the reliability of our findings. Taken together, pooling ADA and IFX is unlikely to have affected microbe-drug interactions; nonetheless, future validation in independent cohorts will be important to replicate these findings.

In this study we present a machine-learning based model to predict success or failure of anti-TNFα therapy in patients with CD. Future studies on cohorts with larger sample size are needed to validate our findings, and possibly reveal microbial signatures during treatment to assess the effect of therapy on the microbiome. *M. gnavus* ASVs were most predictive for non-successful therapy response whereas *Blautia* spp. related to treatment success. *In vitro* assays suggested that *M. gnavus* may interfere with the M2 polarizing effect of anti-TNFα. Eventually, designing predictive tests to target tissue-adherent microbial biomarkers could improve the current clinical practice and inform on personalized treatment strategies for CD patients.

## Data Availability

Raw sequencing data have been deposited in the European Nucleotide Archive (ENA) under accession number PRJEB94054.

## Supplementary material

Supplementary Table 1 Baseline clinical characteristics of patients treated with vedolizumab. Values are median (interquartile range) unless otherwise defined. The number of missing data is shown in square brackets. Percentages have been calculated in the available data. Anti-TNFα: infliximab & adalimumab; HBI, Harvey Bradshaw Index; SES-CD, simple endoscopic disease activity score; Immunomodulator: azathioprine, mercaptopurine, thioguanine, methotrexate.

Supplementary Table 2 Baseline clinical characteristics of patients treated with ustekinumab. Values are median (interquartile range) unless otherwise defined. The number of missing data is shown in square brackets. Percentages have been calculated in the available data. Anti-TNFα: infliximab & adalimumab; HBI, Harvey Bradshaw Index; SES-CD, simple endoscopic disease activity score; Immunomodulator: azathioprine, mercaptopurine, thioguanine, methotrexate.

Supplementary Table 3 Complete list of ASVs identified in colonic and ileal biopsies from Crohn’s disease patients included in this study. ASVs are listed in alphabetical order based on their representative sequences.

Supplementary Table 4 List of ASVs annotated as host-associated eukaryotes or of unknown origin, and therefore excluded from downstream analysis. This table represents a subset of ASVs listed in **Supplementary Table S3**.

Supplementary Table 5 Euclidean distances between the centroids of the three intervention cohorts.

Supplementary Table 6 Alpha diversity metrics in colonic (left) and ileal (right) biopsies from Crohn’s disease patients treated with anti-TNFα, vedolizumab (VDZ), or ustekinumab (USTE). Metrics include Observed richness, Shannon index, Simpson index, and Fisher’s alpha using the amplicon sequence variant (ASV)-level count table. Comparisons between responders and non-responders within each treatment group were performed using the Wilcoxon rank-sum test. Reported values include the W statistic, *P-value*, and 95% confidence interval (CI) of the difference in medians.

Supplementary Table 7 Beta diversity analysis in colonic (left) and ileal (right) biopsies from Crohn’s disease patients treated with anti-TNFα, vedolizumab (VDZ), or ustekinumab (USTE). Principal coordinates analysis (PCoA) was performed based on Bray–Curtis dissimilarity (BCD), unweighted UniFrac distance (UUD), and weighted UniFrac distance (WUD) matrices at amplicon sequence variant (ASV) level. Comparisons between responders and non-responders were assessed using permutational multivariate analysis of variance. Reported values include degrees of freedom (Df), R², F statistic (F-model), and *P-value*.

Supplementary Table 8 Performance metrics of six extra trees-based models (i–vi) predicting treatment response in Crohn’s disease. Columns include: Model (identifier), Sub-cohort (treatment type and biopsy location, e.g., anti-TNFα - colon, VDZ - ileum), Area Under the Curve (AUC) of each model, and *P-value* assessing model significance. *P-values* were calculated by comparing extra trees-based model performance to 200 randomly generated models with permuted response labels to evaluate overfitting.

Supplementary Table 9 List of the 15 most informative ASVs from colonic biopsies distinguishing responders and non-responders to anti-TNFα therapy. This table represents a subset of ASVs listed in **Supplementary Table S3**. Columns include: ASV_ID (amplicon sequence variant identifier), Representative sequence, Feature importance (from the extra trees-based model), SILVA 132 Taxonomy (Family | Genus | Species) assigned by metabarcoding, Higher (indicating association with response or non-response), and BLASTN results including Query cover (%) and Percent identity (%). Manual BLASTN was performed only if genus-level taxonomy was unavailable or needed further confirmation; otherwise, BLASTN columns are left empty.

Supplementary Figure 1 Baseline colonic mucosal-adherent microbiome in patients treated with adalimumab (ADA; n = 13 responders *vs.* n = 10 non-responders) and infliximab (IFX; n = 9 responders *vs.* n = 6 non-responders). Alpha diversity measured by Shannon and Simpson indices at amplicon sequence variant (ASV) level. Wilcoxon signed-rank test. Principal coordinate analysis (PCoA) based on Bray-Curtis dissimilarity at ASV level, illustrating beta diversity between responders and non-responders. Permutational multivariate analysis of variance (PERMANOVA).

Supplementary Figure 2 Differential analysis of the relative abundance of the 15 most predictive ASVs from the anti-TNFα – colon model (**Figure 5B**) between responders and non-responders to adalimumab (ADA; n = 13 responders, n = 10 non-responders) and infliximab (IFX; n = 9 responders, n = 6 non-responders). Comparisons were performed using the Mann-Whitney U test separately for each medication. For ADA, four ASVs showed significantly different relative abundances between responders and non-responders and are displayed in the upper panel. For IFX, no comparisons reached statistical significance; the corresponding dot plots are shown in the lower panel.
