# Supplementary figures and images for: Colonic biopsy-associated microbial signatures are predictive of response to anti-TNFα biological therapy in Crohn’s disease
Source: Front Cell Infect Microbiol. 2026 Mar 4;16:1741002. doi: 10.3389/fcimb.2026.1741002 (PMC12996044; doi:10.3389/fcimb.2026.1741002)

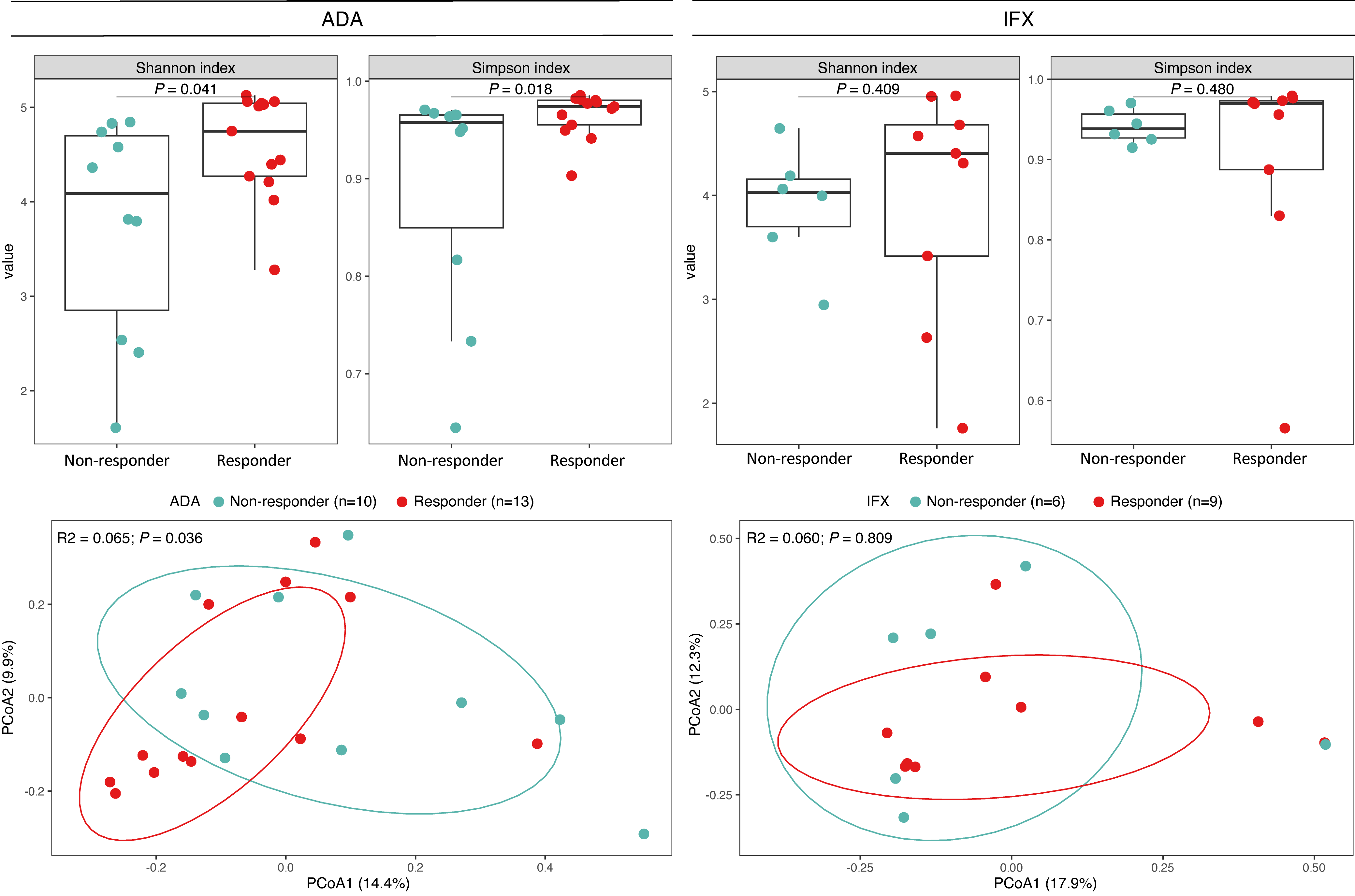

Supplement: Supplementary Figure 1 — Baseline colonic mucosal-adherent microbiome in patients treated with adalimumab (ADA; n = 13 responders vs. n = 10 non-responders) and infliximab (IFX; n = 9 responders vs. n = 6 non-responders). Alpha diversity measured by Shannon and Simpson indices at amplicon sequence variant (ASV) level. Wilcoxon signed-rank test. Principal coordinate analysis (PCoA) based on Bray-Curtis dissimilarity at ASV level, illustrating beta diversity between responders and non-responders. Permutational multivariate analysis of variance (PERMANOVA). [file Image1.tif]

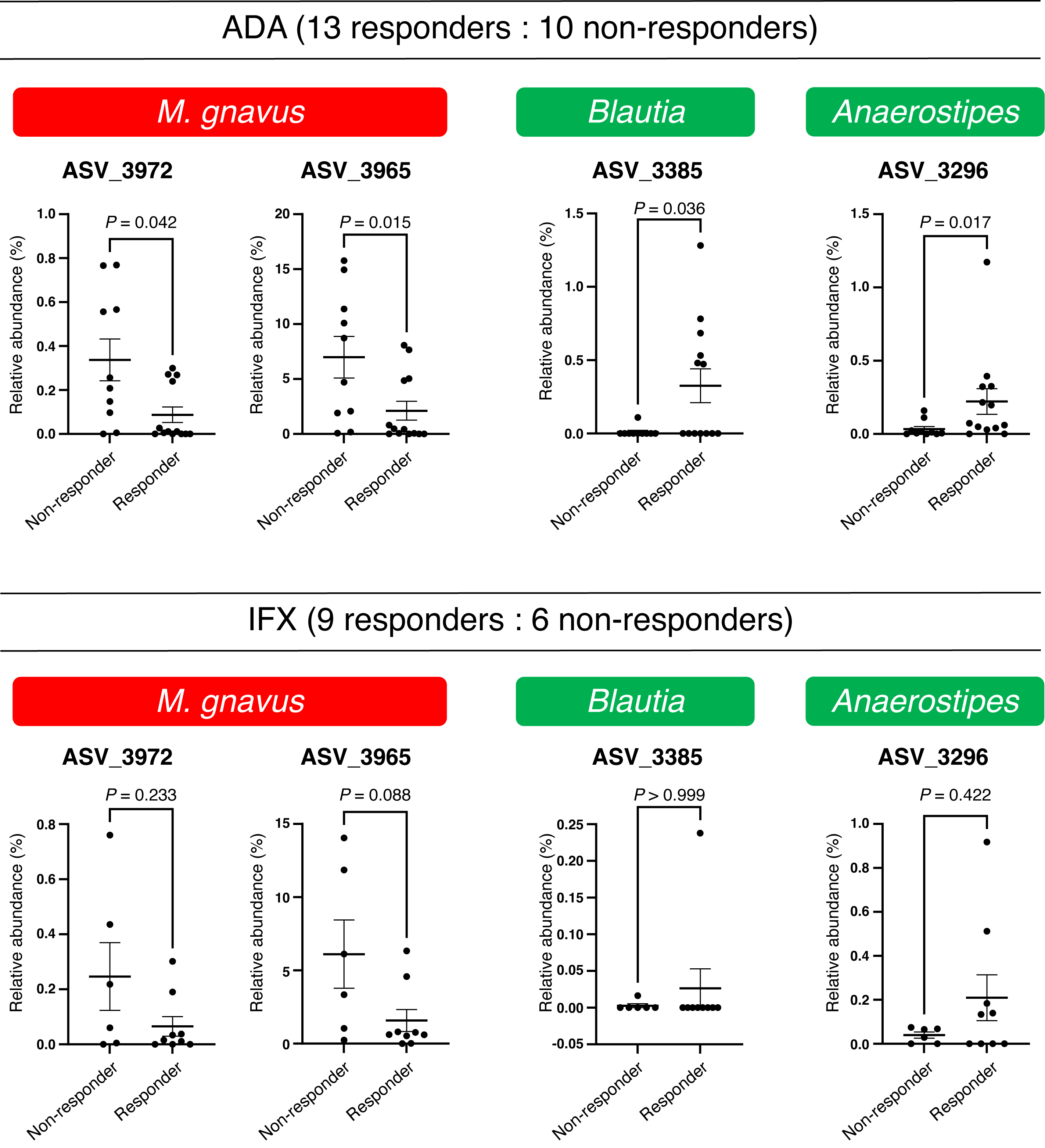

Supplement: Supplementary Figure 2 — Differential analysis of the relative abundance of the 15 most predictive ASVs from the anti-TNFα – colon model (Figure 5B) between responders and non-responders to adalimumab (ADA; n = 13 responders, n = 10 non-responders) and infliximab (IFX; n = 9 responders, n = 6 non-responders). Comparisons were performed using the Mann-Whitney U test separately for each medication. For ADA, four ASVs showed significantly different relative abundances between responders and non-responders and are displayed in the upper panel. For IFX, no comparisons reached statistical significance; the corresponding dot plots are shown in the lower panel. [file Image2.tif]
